# Characterization of Two Ferroptosis Subtypes With Distinct Immune Infiltration and Gender Difference in Gastric Cancer

**DOI:** 10.3389/fnut.2021.756193

**Published:** 2021-12-16

**Authors:** Junfu Ma, Xin Hu, Yanxin Yao, Liuxing Wu, Chao Sheng, Kexin Chen, Ben Liu

**Affiliations:** Key Laboratory of Molecular Cancer Epidemiology of Tianjin, Department of Epidemiology and Biostatistics, National Clinical Research Center for Cancer, Tianjin Medical University Cancer Institute and Hospital, Tianjin, China

**Keywords:** gastric cancer, ferroptosis, subtypes, gender difference, immune infiltration

## Abstract

**Background:** Iron is an essential nutrient involved in the redox cycle and the formation of free radicals. The reprogramming of iron metabolism is the main link to tumor cell survival. Ferroptosis is an iron-dependent form of regulated cell death associated with cancer; the characteristics of ferroptosis in cancers are still uncertain. This study aimed to explore the application value and gender difference of ferroptosis in prognosis and immune prediction to provide clues for targeted therapy of gastric cancer.

**Methods:** We comprehensively evaluated the ferroptosis levels of 1,404 gastric cancer samples from six independent GC cohorts based on ferroptosis-related specific genes and systematically correlated ferroptosis with immune cell infiltrating and gender characteristics. The ferroptosis score was constructed to quantify the ferroptosis levels of individual tumors using principal component analysis (PCA) algorithms.

**Results:** We identified two distinct ferroptosis subtypes in gastric cancer, namely Subtype-A and Subtype-B. We found that male patients in Subtype-B had the worst prognosis in contrast with the other groups. Three sex hormone receptors (AR, ER, and PR) in Subtype-B tumor patients were higher than in Subtype-A tumor patients in GC, while the HER2 displayed an opposite trend. We developed a risk model termed ferroptosis score to evaluate ferroptosis levels within individual tumors. The low-ferroptosis score group was characterized by activation of immune cells and increased mutation burden, which is also linked to increased neoantigen load and enhanced response to anti-PD-1/L1 immunotherapy. The patients with a low-ferroptosis score showed a high microsatellite instability status (MSI-H) and had a higher response to immunotherapy. Furthermore, the patients with low-ferroptosis scores have a lower estimated IC50 in the several chemotherapy drugs, including paclitaxel, gemcitabine, and methotrexate.

**Conclusions:** We revealed that sex hormone receptors and immune cell infiltration were markedly different between ferroptosis subtypes in GC patients. The results suggested that gender difference may be critical when the ferroptosis-related strategy is applied in GC treatment. Further, ferroptosis levels were identified with an extreme variety of prognosis and tumor immune characteristics, which might benefit GC individualized treatment.

## Introduction

Gastric cancer (GC) is the fifth most commonly diagnosed cancer globally and the third leading cause of cancer-related deaths (1). The incidence of gastric cancer shows a mysterious male advantage with a male-to-female ratio of about 2:1, and women have a higher survival rate after treatment (1, 2). Although significant efforts have been devoted to treating GC, effectively individualized therapeutic strategies remain to be explored (3, 4).

Iron is the most abundant essential trace element in the human body, and it is also considered indispensable for cancer development. As an essential nutrient to promote cell proliferation and growth, the reprogramming of iron metabolism is the main link to tumor cell survival (5). Ferroptosis, an iron-dependent form of regulated cell death (RCD) driven by the lethal accumulation of lipid peroxidation, has been related to various types of tumors' occurrence and therapeutic response (6–8). In recent years, ferroptosis induction has become a promising treatment alternative to trigger cancer cell death, especially for those aggressive malignant tumors resistant to traditional therapies (9, 10). Ferroptosis can be induced through extrinsic or intrinsic pathways (11). The extrinsic pathway is initiated through the Regulation of transporters. In contrast, the intrinsic pathway is mainly caused by blocking the expression or activity of intracellular antioxidant enzymes, such as glutathione peroxidase 4 (GPX4). The antioxidant enzyme GPX4 can directly reduce phospholipid hydroperoxide to a hydroxy phospholipid, thus acting as a central repressor of ferroptosis in cancer cells (12).

Immune checkpoint blocking therapy has been demonstrated to improve survival across multiple tumor types, including GC (13–15). The tumor mutation burden (TMB), microsatellite instability (MSI) status, and PD-L1 expression were reported as potential biomarkers that favor PD-1 blockade-based immunotherapy (16). Growing evidence implies that targeting iron metabolic pathways and inducing ferroptosis may provide new tools for treating drug-resistant cancers. For example, Yi et al. found that cancer cells often show metabolic changes that make them vulnerable to ferroptosis, a particular type of cell death. It was also found that the combination of drugs that blocking the PI3K-AKT-mTOR pathway and induce ferroptosis could significantly destroy and clear the tumor (17). The long-term effects of ferroptosis on tumor immunity depend on the interaction between cancer cells and various immune cell subpopulations. For example, the lymphatic system protects melanoma cells from ferroptosis by increasing ACSL3-dependent production of monounsaturated fatty acids (MUFAs), promoting tumor metastasis (18). In addition, despite subtle indications that the potential level of ferroptosis varies between the sexes in several cancers (19), few studies consider gender bias in gastric cancer when using ferroptosis as a treatment strategy. Therefore, more exploration of the ferroptosis heterogeneity might be helpful for individual and accurate treatment in GC.

This study collected 1,404 GC samples from six independent GC cohorts for analysis and identified two subtypes. We surprisingly found that the two subtypes were highly inconsistent in gender characteristics and immune cell infiltration. For that, we established a set of scoring systems to quantify the ferroptosis levels in individual patients and dissected the relations between ferroptosis and biological features. It was found that ferroptosis was associated with various cancer hallmarks, hormone receptors, immune infiltration, drug sensitivity, and patient survival. These results emphasize the critical role of ferroptosis in GC and contribute to the further investigations of ferroptosis-related molecular mechanisms and treatment development.

## Materials and Methods

### Data Acquisition

Public gene-expression data and complete clinical information were obtained through Gene Expression Omnibus (GEO, https://www.ncbi.nlm.nih.gov/geo) and the Cancer Genome Atlas (TCGA, https://portal.gdc.cancer.gov/) database. Patients without survival information were removed from further evaluation. In total, five eligible GC cohorts [GSE15459, GSE34942, GSE62254/ACRG, GSE84437, TCGA-STAD (The Cancer Genome Atlas-Stomach Adenocarcinoma)] were gathered in this study for further analysis. Gene expression data and the clinical data of 90 Tianjin GC samples were obtained from previous studies (20). In short, six cohorts, including 1,404 samples, were integrated into the study as a training set. In addition, GSE26899 and GSE26901 datasets were included in the study as two independent validation cohorts. The baseline information of all eligible GC datasets was summarized in Supplementary Table S1. Ferroptosis-related genes (FRGs) were identified from published literature (21).

### Identification of Differentially Expressed Genes

Unsupervised clustering analysis was conducted to identify distinct clusters according to the expression of 60 ferroptosis-related genes. We used the R package ConsensusClusterPlus (v 1.58.0) to execute the consensus clustering algorithm and repeat it 1,000 times to ensure the clustering stability (22).

Differentially expressed genes (DEGs) (Supplementary Table S2) between different clusters were determined using the empirical Bayesian approach of the limma (v 3.50.0) R package (23). The significance criterion for determining DEGs was |log fold change (FC)| > 1 and adjusted *P*-value < 0.05. The Benjamini and Hochberg method was applied for multiple-testing adjustments.

### Identification of the Ferroptosis Subtypes in Gastric Cancer

First, using univariate Cox regression analysis, selected 138 differentially expressed genes with prognostic value (Supplementary Table S3) for further analysis (*P* < 0.01). We named these 138 genes as “subtype-specific genes.”

Next, we performed unsupervised clustering of all samples using the expression values of 138 subtype-specific genes via the R package “ConsensusClusterPlus.” Two ferroptosis subtypes were obtained, named “Subtype-A” and “Subtype-B.”

### Gender Difference Analysis Among Subtypes

Patients in the two subtypes were stratified according to gender, and the Kaplan-Meier survival analysis was used to assess the gender differences in overall survival (OS). The log-rank test was used to evaluate the statistical significance of the Kaplan-Meier curves at *P* < 0.05.

To further explore the factors of gender differences between the two ferroptosis subtypes, we analyzed the expressions of three sex hormone receptors (AR, ER, PR) and HER2 of the two subtypes using the Wilcoxon rank-sum test. Furthermore, we stratified the two subtypes by gender and compared their differences in hormone receptor levels, respectively.

### Significantly Mutated Genes Landscapes and Mutation Patterns in the Two Subtypes

We recognized the significantly mutated genes (SMG) with the GenVisR (v 1.26.0) tool in the R package. Mutation signature analysis of two ferroptosis subtypes was conducted using the R package MutationalPatterns (v 3.4.0) and maftools (v 2.10.0). We extracted the mutational signature of GC data and compared them with the mutation database (COSMIC V2) by using the cosine similarity method (https://cancer.sanger.ac.uk/cosmic).

### Pathway Enrichment Analysis

Gene set variation analysis (GSVA) (24) was performed with the R package “GSVA”(v 1.42.0) to evaluate pathway enrichment for two ferroptosis subtypes. All hallmark gene sets were downloaded from the Molecular Signature Database (MSigDB). Adjusted *P* with a value < 0.05 was considered statistically significant. The Benjamini and Hochberg method was applied for multiple-testing adjustments.

### Estimation of Immune Cell Infiltration

The single-sample gene set enrichment analysis (ssGSEA) (25) was introduced to quantify the relative infiltration of 28 immune cell types in the tumor microenvironment. Unique feature gene panels for each immune cell subset were obtained from the latest literature (26). An enrichment score in ssGSEA analysis represented the relative abundance of each immune cell type. The ssGSEA score was normalized to unity distribution, for which zero is the minimal and one is the maximal score for each immune cell type. The bio-similarity of the immune cell filtration was estimated by multidimensional scaling (MDS) and a Gaussian fitting model.

In addition, we assessed the abundances of tumor immune infiltration in subpopulations of M0 and M1 macrophages using the CIBERSORT algorithm. Then, the infiltration abundance of M2a, M2b, M2c, and M2d macrophage subpopulations was analyzed using the ssGSEA algorithm based on four subtype-related markers (27).

### Generation of the Ferroptosis-Related Risk Model

Principal component analysis (PCA) was conducted to construct the ferroptosis relevant risk score, which we called was ferroptosis score. Principal components 1 and 2 were selected as signature scores. The advantage of this method is that the score is concentrated on the set with the largest block of well-correlated (or anticorrelated) genes in the set, while down-weighting contributions from genes that do not track with other set members (28, 29):

ferroptosis score = ∑(PC1_i_ + PC2_i_)

where i is the expression of subtype-specific genes.

The GC samples were categorized into high- and low-ferroptosis score groups according to the optimal cut-off value determined by the survminer R package. Then, we performed Kaplan-Meier analysis of ferroptosis score in GC cohort (*n* = 1,404) and calculated the Hazard Ratio (HR) by univariate and multivariate cox regression analysis to further assess the predictive accuracy of the model. Furthermore, we did external validation in two independent cohorts: GSE26899 (*n* = 93) and GSE226901 (*n* = 109). We fitted the ferroptosis score with the meaningful clinical indicators obtained by multivariate cox regression, and the accuracy of the ferroptosis score was evaluated using the receiver operating characteristic (ROC) curves. The R package “glmnet” was used for Logistic regression analysis for multivariate fitting, and the R package “timeROC” was used to draw ROC curves.

To investigated the association between ferroptosis score and biological factors, we performed correlation analysis to reveal the association between ferroptosis score and AR, ER, PR, HER2, and 28 immune cell types.

### Immunotherapy and Chemotherapy Response With Ferroptosis Score

We compared three remarkable molecular biomarkers, including TMB, MSI, and TIDE, between high- and low-ferroptosis score groups. Microsatellite instability (MSI) status was classified as Microsatellite stable (MSS), MSI-low (MSI-L, one marker unstable), and MSI-high (MSI-H, over two markers unstable). The data of MSI status were obtained from the UCSC Xena database.

In order to validate the efficiency of the ferroptosis score in males and females, an immunotherapeutic cohort was included in our study: advanced urothelial cancer with the intervention of atezolizumab, an anti-PD-L1 antibody (IMvigor210 cohort) (30). Clinical information and gene expression data were extracted from the IMvigor210 data set (http://research-pub.gene.com/IMvigor210CoreBiologies).

The R package “pRRophetic” (v 0.5) (31) was used to predict chemotherapeutic response in GC patients. The half-maximal inhibitory concentration (IC50) of the samples was demonstrated using ridge regression, and the prediction accuracy was assessed using 10-fold cross-validation based on the GDSC training set (32).

### Statistical Analysis

Clustering of ferroptosis was performed using the consensus clustering method to identify structure over many clustering realizations robustly (33, 34). The survival curves for the prognostic analysis were generated via the Kaplan-Meier method, and log-rank tests were used to identify the significance of differences. Univariate and multivariate Cox regression models were employed for calculating hazard ratio (HR) and forest plots visualized the coefficients of those regression models. Continuous variables were compared between two groups through was performed by Wilcoxon rank-sum test. Classified variables were compared between two groups through was performed by chi-square test. Kruskal-Wallis test was used to conduct difference comparisons of three or more groups. Correlations between variables were assessed via Pearson and Spearman coefficient. Differences were considered statistically significant when a *P*-value was < 0.05, and all *P*-values reported were two-sided. All statistical analyses were conducted using R 4.0.0 (https://www.r-project.org/).

## Results

### Identification of the Ferroptosis Subtypes

**Figure 1:**
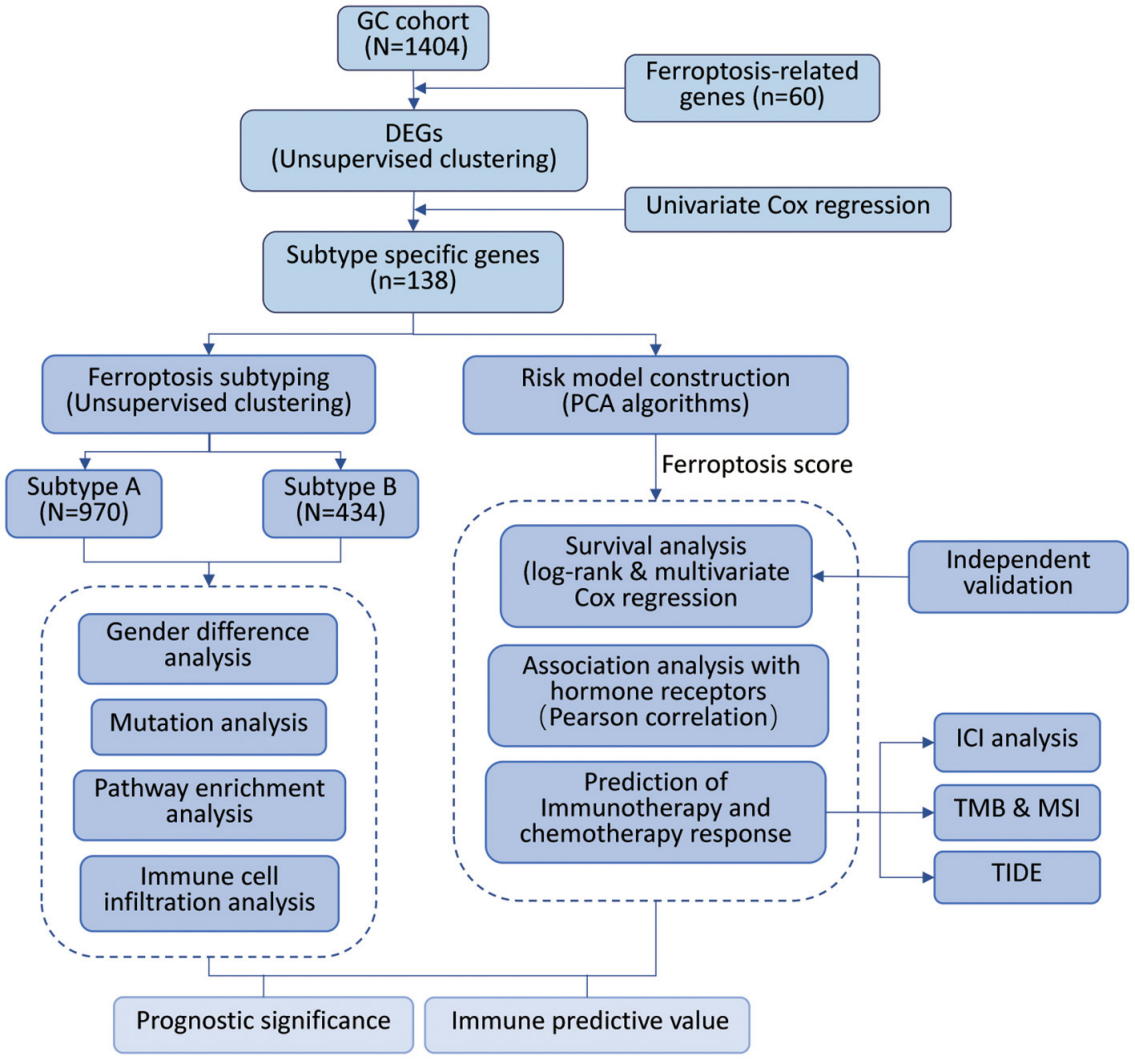
Overview of study design and analysis of ferroptosis.

To systematically describe our study, we developed a flow chart (Figure 1). A total of 1,404 samples from six independent GC cohorts accompanied with complete survival information were retained for the present study. All GC cases were divided into two clusters by unsupervised clustering of the expression of 60 ferroptosis-related genes (FRGs). Survival analysis indicated a difference in prognosis between the two clusters (log-rank test, *P* = 0.039; Supplementary Figure S1).

**Figure 2:**
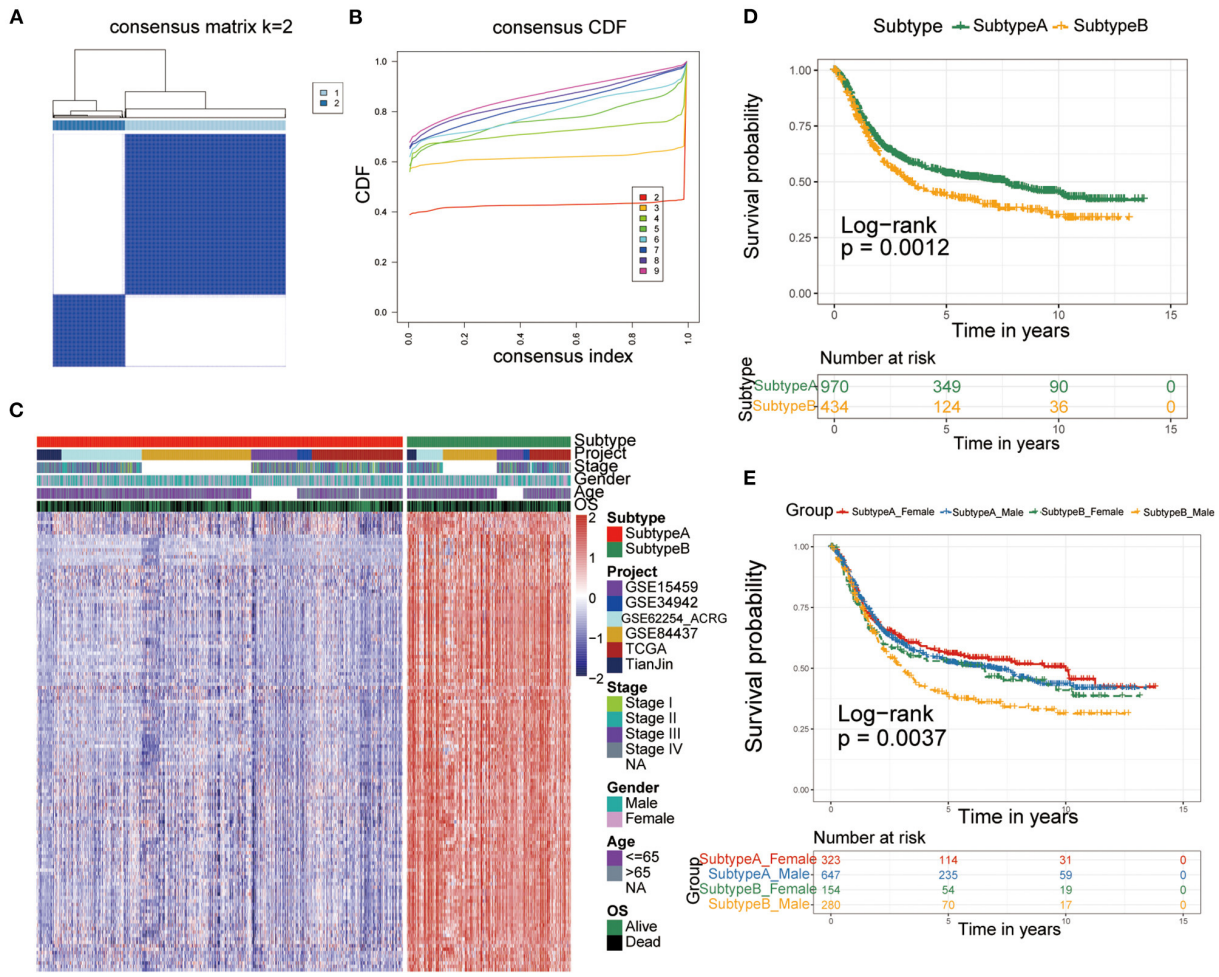
Identification of ferroptosis subtypes. **(A,B)** The optimal number of clusters (K = 2) was determined from cumulative distribution function (CDF) curves, and the classification effect is the best. **(C)** Heatmap of the expression of 138 subtype-special genes. **(D)** Kaplan-Meier curves for survival prediction of patients in the two subtypes. **(E)** Kaplan-Meier curves for survival prediction of patients in the two subtypes after stratification by gender.

To construct a more precise ferroptosis subtype with prognostic significance, we recognized 278 differentially expressed genes (DEGs) among clusters and certified 138 DEGS with prognostic value by univariate Cox regression analysis. Subsequently, we carried out secondary clustering that the consensus cluster analysis indicated that the optimal number of clusters was two, defined by CDF curves (Figures 2A,B). Unsupervised clustering analysis based on the expression of these 138 genes also divided GC patients into two clusters, which we called Subtype-A and Subtype-B.

Therefore, the 138 genes are called “subtype-specific genes” (Figure 2C).

Additionally, survival analysis indicated that Subtype-A had a better prognosis (log-rank test, *P* = 0.0012; Figure 2D). Considering that there may be gender differences in individual ferroptosis levels (19), we stratified the two subtypes by sex and conducted a survival analysis to observe the prognosis. As shown in Figure 2E, male patients with gastric cancer had the worst prognosis in Subtype-B (log-rank test, *P* = 0.0037).

### Molecular Characteristics Related to Gender Between Ferroptosis Subtypes

**Figure 3:**
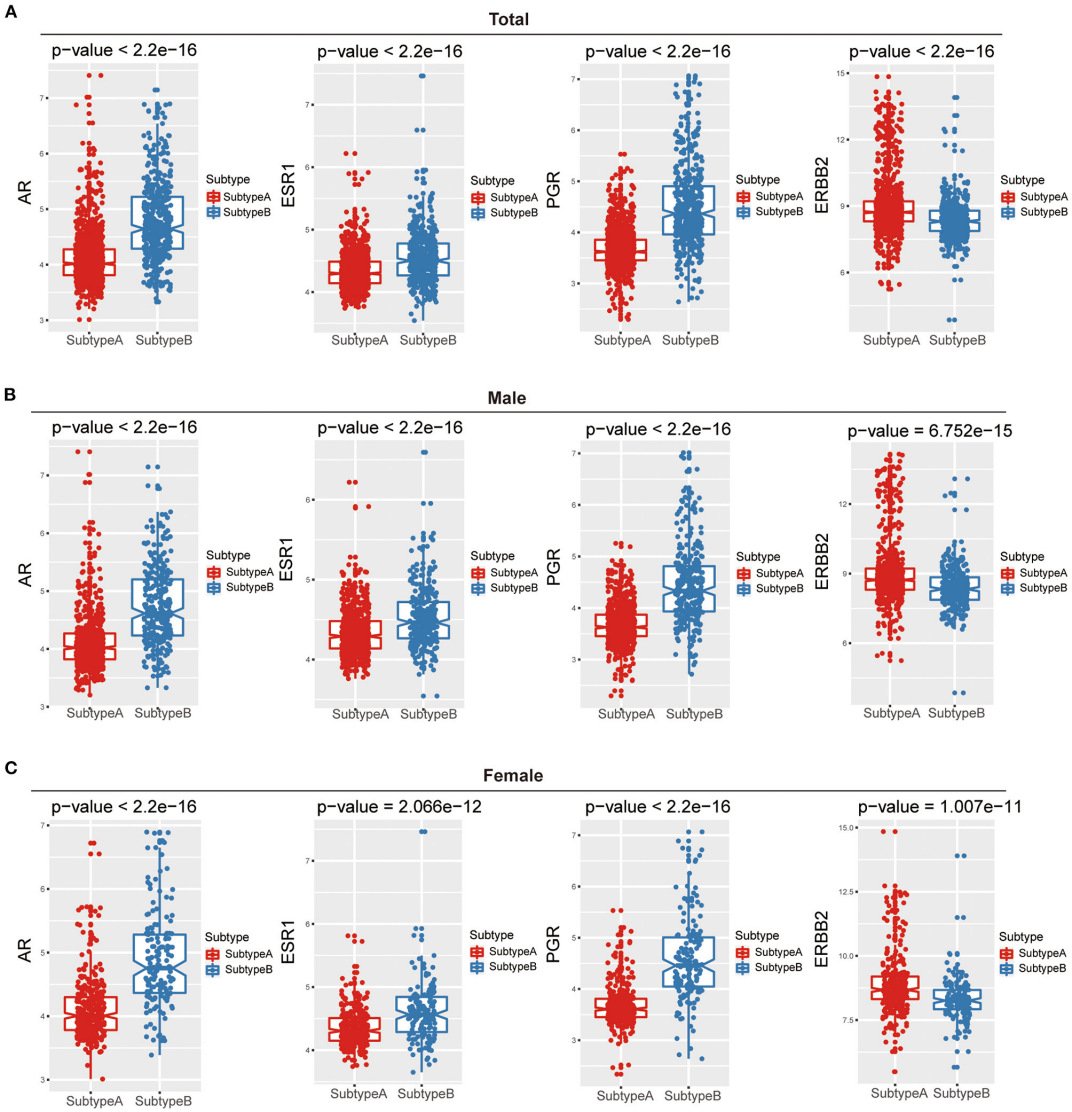
The relations between hormone receptor and ferroptosis subtypes among gender. Box plots show that the difference in hormone receptors between two ferroptosis subtypes of total **(A)**, male **(B)**, and female **(C)**.

To further dissect the factors involved in gender differences, we examined three sex hormone receptors (AR, ER, and PR) and HER2 between Subtype-A and Subtype-B. The results presented in Figure 3A showed that all hormone receptors differ significantly between the two subtypes (Wilcoxon rank-sum test, all *P* < 2.2e-16).

Furthermore, we compared three sex hormone receptors (AR, ER, and PR) and HER2 between the two subtypes in males and females, respectively. The results were consistent with the results of the above overall analysis, and there were significant differences in the four hormone receptors among the two ferroptosis subtypes in both males and females (Figures 3B,C).

### Analysis of Mutation Pattern in Distinct Ferroptosis Subtypes

**Figure 4:**
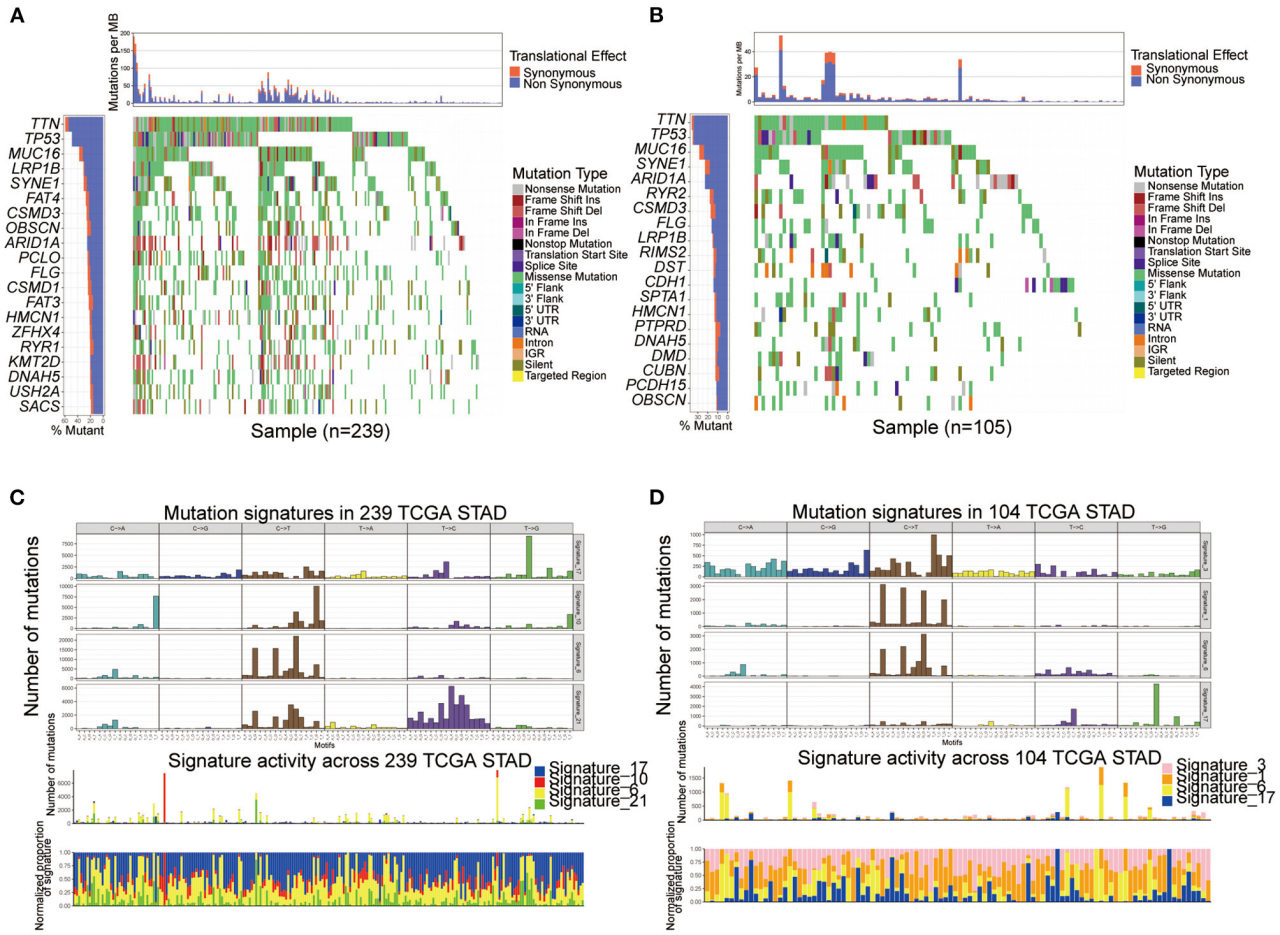
Comparison of mutational patterns and signatures in the two ferroptosis subtypes of TCGA GC samples. **(A,B)** The waterfall plot of tumor somatic mutation was established by those with Subtype-A **(A)** and Subtype-B **(B)**. **(C,D)** Mutation signature extracted in the Subtype-A **(C)** and Subtype-B **(D)**.

We performed SMG analysis for GC samples in Subtype-A and Subtype-B. The SMG mutational landscapes of these two subgroups (Figures 4A,B) exhibited a distinct mutation ratio, and the top three were the same between subtypes: TTN (59.4 vs. 36.2%), TP53 (49.0 vs. 35.2%), and MUC16 (37.7 vs. 28.6%). The fourth place in Subtype-A was LRP18 (31.0%), while in Subtype-B was SYNE1 (22.9%).

To gain further insights into the operative mutational processes in the two subtypes, we extracted the mutational signatures from the COSMIC database using GC's genomic somatic mutation data. We extracted four mutational signatures (i.e., signatures 17, 10, 6, and 21; Figure 4C) from mutation data of Subtype-A. We extracted four mutational signatures (i.e., signatures 3, 1, 6, and 17; Figure 4D) from mutation data of Subtype-B. Subtype-A had the independent characteristics of signature 10 and signature 21, while Subtype-B had the independent characteristics of signature 3 and signature 1. These results suggested that the mutation pattern of Subtype-B was associated with DNA damage and repair pathways such as homologous recombination, leading to DNA double-strand break repair failure.

### The Difference in Pathway Enrichment and Immune Infiltration Between Ferroptosis Subtypes

**Figure 5:**
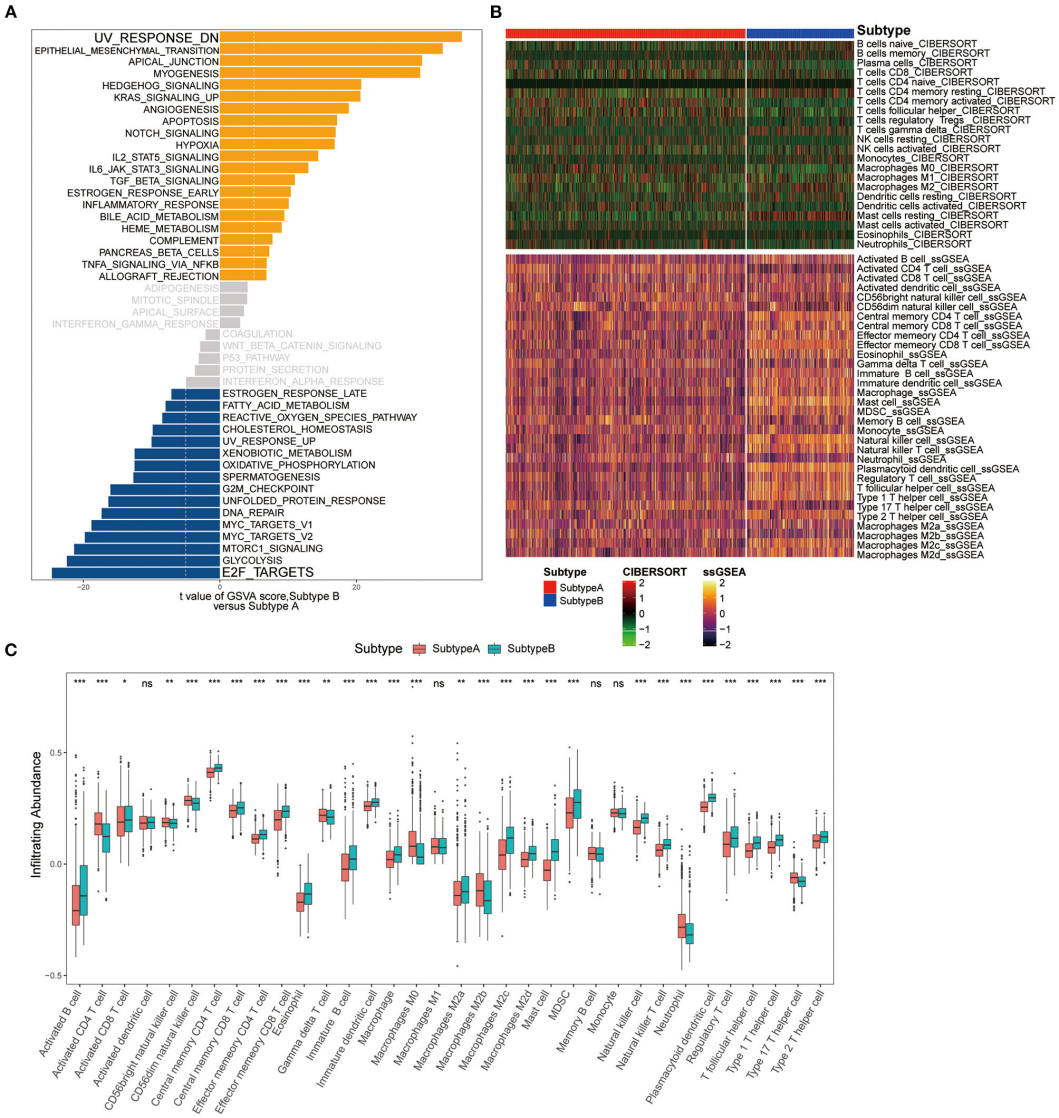
Pathway enrichment analysis and immune cell infiltration among subtypes. **(A)** Enrichment analysis for metabolism pathway between Subtype-A and Subtype-B. **(B)** Heatmap shows the landscape of immune infiltration based on CIBERSORT and ssGSEA algorithms. **(C)** Box plot shows the difference in immune cell infiltration between two ferroptosis subtypes.

To explore the differences in biological behaviors between the two subtypes, we performed a GSVA enrichment analysis. As shown in Figure 5A, the two subtypes showed some differences, E2F_TARGETS as the top enriched signature in Subtype-A and UV_RESPONSE_DN as the top enriched signature in Subtype-B. The results from GSVA analyses have revealed Subtype-B was significantly associated with genes down-regulated in response to ultraviolet (UV) radiation, Subtype-A was significantly associated with genes encoding cell cycle-related targets of E2F transcription factors. Therefore, we speculated that Subtype-B has more obvious DNA damage.

We next composed a heatmap to visualize the relative abundance of immune infiltrating cell subpopulations from the discovery dataset (Figure 5B). We found a significant difference in immune cell infiltration between the two subtypes (Figure 5C). In addition, it can be observed that there are differences in immune infiltration among macrophage subpopulations of ferroptosis subtypes. There was no difference in M1 macrophage abundance between the two subtypes. M0 and M2b subpopulations were significantly enriched in the Subtype-A (both *P* < 0.001), while M2a (*P* < 0.01), M2c (*P* < 0.001), and M2d (*P* < 0.001) subpopulations were significantly enriched in the Subtype-B.

### Risk Model Construction and Ferroptosis Score

To better predict the prognosis and guide individualized treatment, we construct a ferroptosis-related risk model. The ferroptosis score was used to quantify the ferroptosis levels of individual patients with gastric cancer, considering the individual heterogeneity and complexity of gene expression levels. The ferroptosis score was modeled based on the expression of subtype-specific genes by PCA algorithms. Supplementary Figure S2 visualizes the contribution of two components of the PCA algorithm.

**Figure 6:**
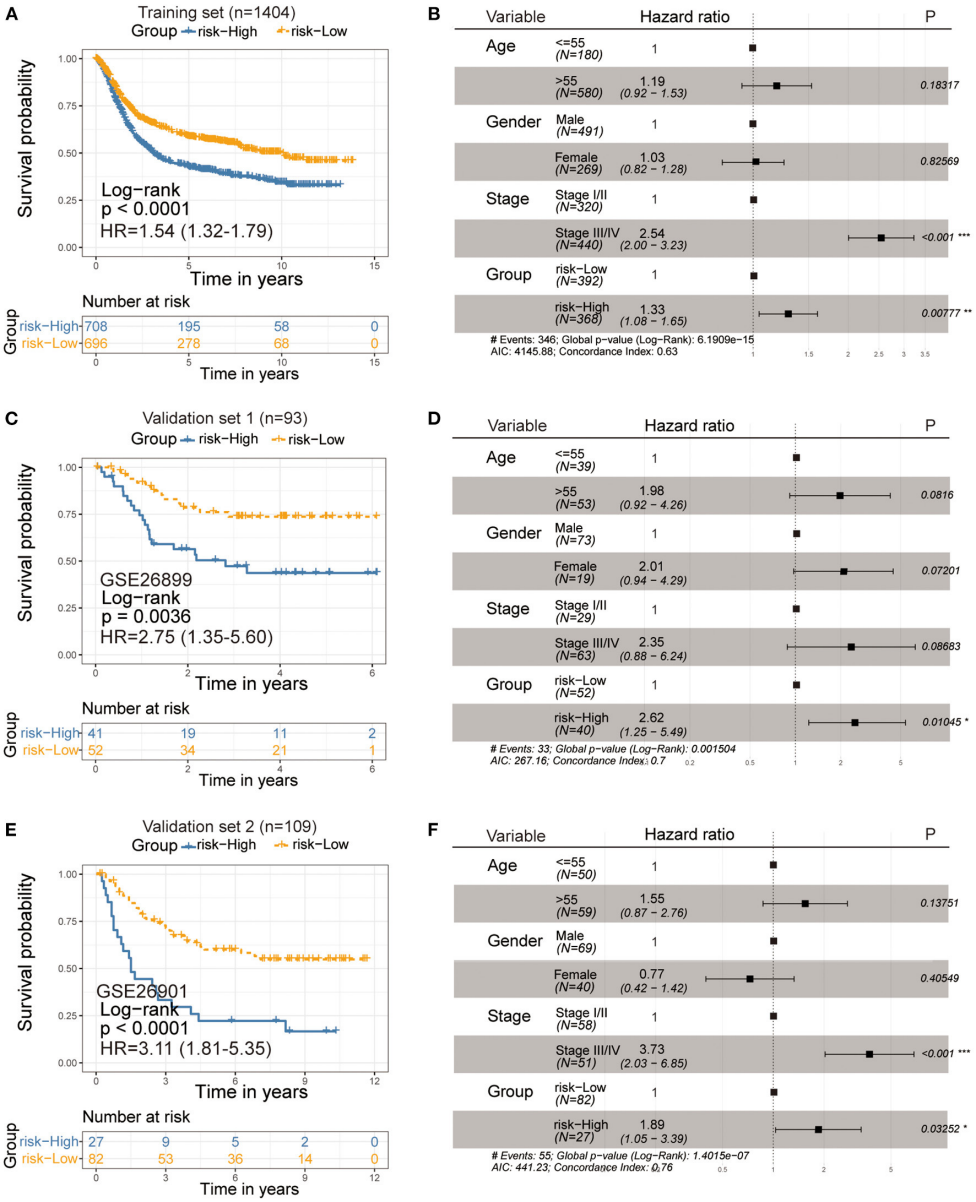
Kaplan-Meier curves for survival prediction in high- and low-ferroptosis score groups of gastric cancer (Left). Forest plots representation of the multivariate Cox regression analysis of ferroptosis score with age, gender, tumor stage was taken into account (Right). **(A,B)** Training set-GC cohort, **(C,D)** Verification set-GSE26899, **(E,F)** Verification set-GSE26901. *P-*values are from the log-rank test. HR with 95% CI was from univariate and multivariate cox regression analysis.

Next, we sought to identify the value of the ferroptosis score in predicting the outcome of GC patients. Patients were divided into high- and low-ferroptosis score groups with the optimal cut-off value determined by the survminer R package. We performed Kaplan-Meier survival analysis and univariate cox regression analysis, and the result showed that patients with low-ferroptosis scores demonstrated a prominent survival benefit [log-rank test, *P* < 0.0001; HR 1.54 (1.32-1.79); Figure 6A]. So “low-ferroptosis score” means “risk-low.” We then tested whether the ferroptosis score could serve as an independent prognostic biomarker for gastric cancer. Multivariate Cox regression analysis, which included the factors of patients' age, gender, tumor stage, confirmed ferroptosis score is an independent prognostic indicator of evaluating patient outcomes [HR 1.33 (1.08-1.65); Figure 6B]. To further test the stability of the risk model, the predictive value was externally validated in the GSE26899 and GSE26901 datasets. Consistent with the training set, the survival analysis results and univariate and multivariate cox regression analysis of GSE26899 (Figures 6C,D) and GSE26901 (Figures 6E,F) datasets were all statistically significant. Hence, the ferroptosis score could be an independent prognostic factor for gastric cancer. ROC curve showed that the ferroptosis score in our present study exhibited a good or acceptable predictive value in the aspect of 1 year AUC, 3 years AUC, and 5 years AUC, which was 0.68, 0.67, and 0.67, respectively (Supplementary Figure S3A). Moreover, in the two verification sets, the ROC curve also validated a good predictive value in the survival time of 1 year, 3 years, or 5 years (Supplementary Figures S3B,C).

### Ferroptosis Score Was Highly Associated With Hormone Receptors and Immune Cell Infiltration

**Figure 7:**
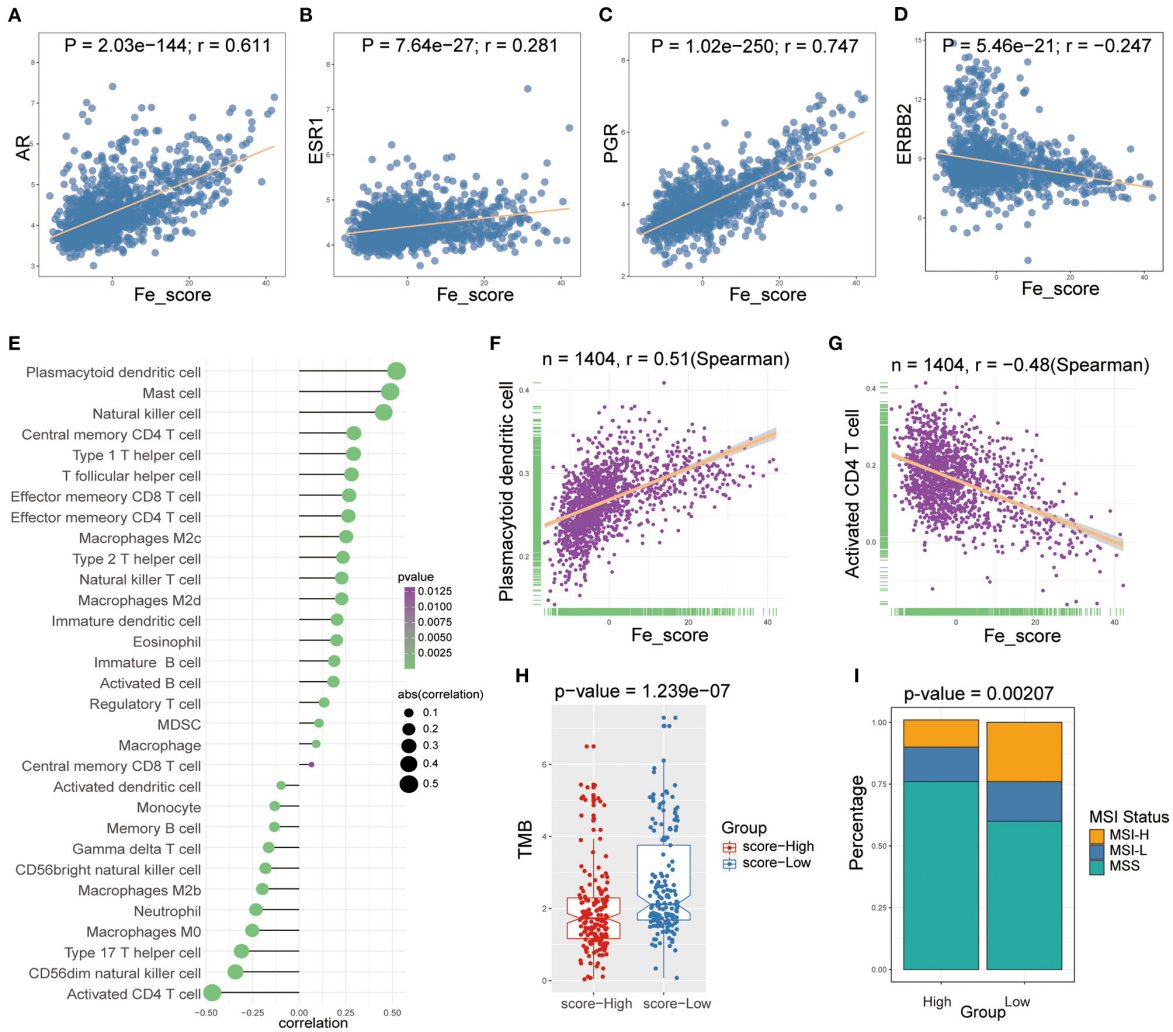
The relations between ferroptosis score and hormone receptors and immune cell infiltration. **(A-D)** The Pearson correlation between ferroptosis score and AR **(A)**, ER **(B)**, PR **(C)**, and HER2 **(D)**. **(E)** Lollipop plot shows relationships between ferroptosis score and 28 kinds of immune cells. **(F,G)** The Spearman correlation between ferroptosis score and the plasmacytoid dendritic cell **(F)** and the activated CD4 T cell **(G)**. **(H)** Box plot was used to visualize the differential tumor mutational burden (TMB) of the high- and low-ferroptosis score groups in GC patients. **(I)** The percent bar chart was used to visualize the differential microsatellite instability (MSI) status of the high- and low-ferroptosis score groups in GC patients.

We examined the specific correlation between ferroptosis score and four hormone receptors using Pearson correlation analyses. The correlation analysis revealed that the ferroptosis score was significantly associated with three sex hormone receptors (AR, ER, PR) and HER2. In detail, ferroptosis score was positively associated with AR (r = 0.611, *P* = 2.03e-144; Figure 7A), ER (r = 0.281, *P* = 7.64e-27; Figure 7B), PR (r = 0.747, *P* = 1.02e-250; Figure 7C), and negatively associated with HER2 (r = −0.247, *P* = 5.46e-21; Figure 7D).

Then, we performed Spearman correlation analysis to explore the relationship between ferroptosis score and immune cell infiltration. As shown in Figure 7E, there was a widespread correlation between the ferroptosis score and 27 out of 28 kinds of immune cell types. And we further analyzed the correlation between ferroptosis score and macrophage subpopulations. Ferroptosis score was positively correlated with M2c and M2d macrophages and negatively correlated with M2b and M0 macrophages. Moreover, we found that the strongest positive correlation was the plasmacytoid dendritic cell (r = 0.51, *P* < 0.0001; Figure 7F), and the strongest negative correlation was the activated CD4 T cell (r = −0.48, *P* < 0.0001; Figure 7G).

### Ferroptosis Score in the Role of Immunotherapy and Chemotherapy

**Figure 8:**
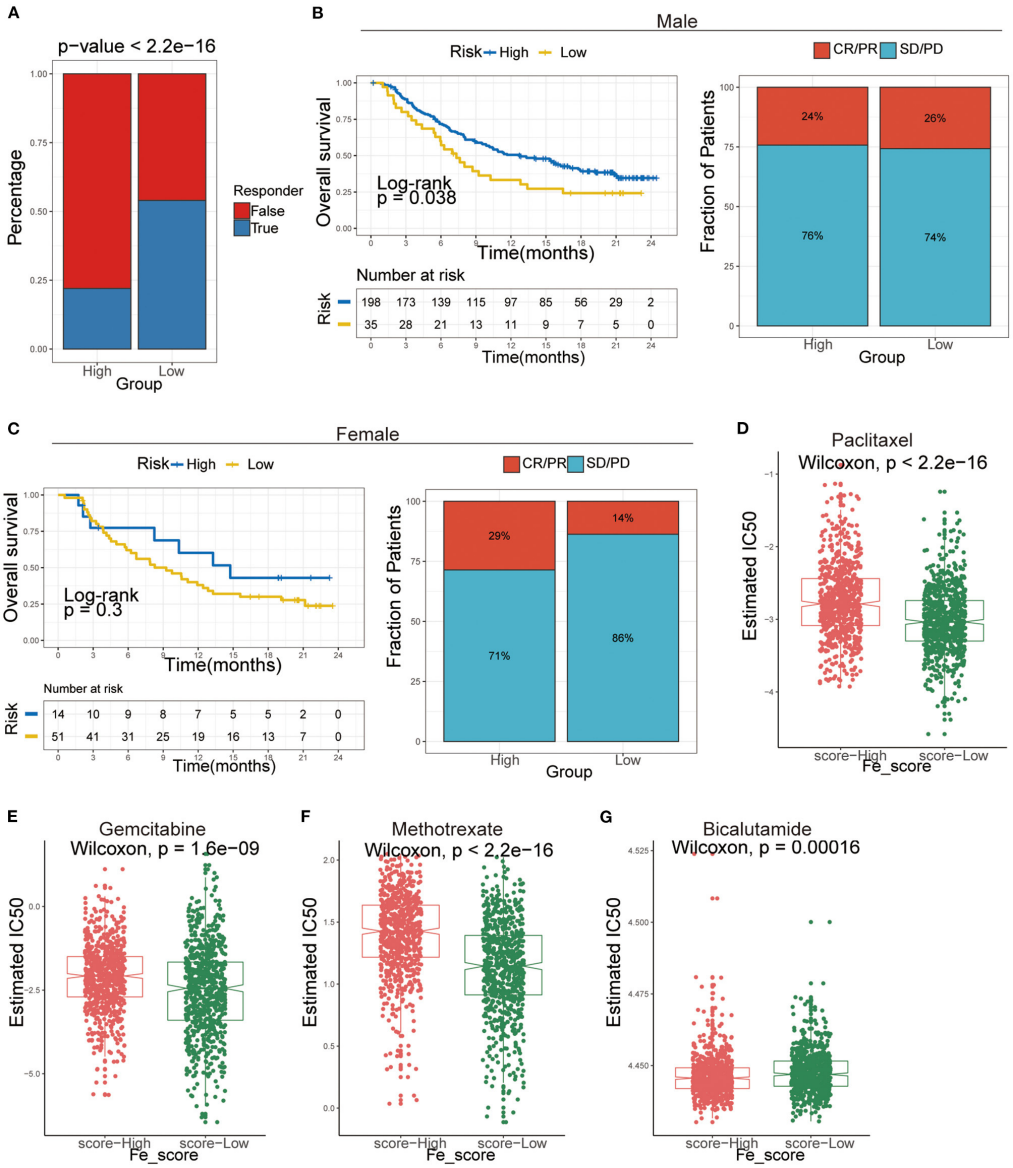
The application of ferroptosis score during the courses of cancer immunotherapy and chemotherapy. **(A)** Tumor Immune Dysfunction and Exclusion (TIDE) analysis predicted the immunotherapy response of high- and low-ferroptosis score groups in GC patients. **(B,C)** Kaplan–Meier analysis and the proportion of immune response to immunotherapy of high- and low-ferroptosis score groups in the UC cohort. **(B)** males; **(C)** females. CR, complete response; PR, partial response; SD, stable disease; PD, progressive disease. **(D-G)** The IC50s of chemotherapeutic agents with ferroptosis score. **(D)** paclitaxel, **(E)** bicalutamide, **(F)** gemcitabine, **(G)** methotrexate.

We analyzed intrinsic immune escape mechanisms between high-and low-ferroptosis score groups in GC patients. The results showed that TMB was relatively higher in the low-ferroptosis score group (Wilcoxon rank-sum test, *P* = 1.239e-07; Figure 7H), indicating the relatively high immunogenicity. Meanwhile, the low-ferroptosis score group tended to have more MSI-H status (chi-square test, *P* = 0.00207; Figure 7I). The above results suggested that patients with low ferroptosis scores were more suitable for immunotherapy. Immunotherapy has manifested improved survival in treating multiple tumor types, and it is urgent to identify patients who will benefit most. Thus, we used the TIDE algorithm to further explored immunotherapy response prediction (35). The results showed that the low-ferroptosis score group had a better response to immunotherapy (Figure 8A). The result was consistent with those of TMB and MSI.

Afterward, we included an immunotherapy cohort to investigate whether ferroptosis scores predicting the patients' responsiveness to immunotherapy differ in males and females. The results showed that males are more sensitive to ferroptosis scores and more likely to benefit from immunotherapy (Figures 8B,C).

Additionally, we performed a prediction analysis of response to chemotherapy in the two groups by applying the pRRophetic R package. Patients in the low-ferroptosis score group had a higher sensitivity for the following chemotherapy drugs: paclitaxel (Wilcoxon rank-sum test, *P* < 2.2e-16; Figure 8D), gemcitabine (Wilcoxon rank-sum test, *P* = 1.6e-09; Figure 8E), methotrexate (Wilcoxon rank-sum test, *P* < 2.2e-16; Figure 8F). Patients in the high-ferroptosis score group had a higher sensitivity for bicalutamide (Wilcoxon rank-sum test, *P* = 0.00016; Figure 8G).

The Asian Cancer Research Group (ACRG) divides GC into four subtypes using gene expression data: microsatellite instability (MSI), epithelial-to-mesenchymal transition (EMT), MSS/TP53+ and MSS/TP53– (36). The subsets described by ACRG were associated with distinct genomic alterations, survival outcomes, and postoperative recurrence patterns (36). Nevertheless, so far, the possibility of GC molecular classification in daily practice and therapeutic implementation is still challenging (37). Our study found no ferroptosis score specificity among ACRG's GC molecular subtypes (Supplementary Figure S4), indicating that our score system differed from ACRG's subtype classification system. It may also mean that better treatment and prognosis can be suggested, but more exploration is needed in the future. Overall, our study strongly confirmed that the ferroptosis score could be used to assess patients' prognosis and immunotherapy response and may be different for males and females. Our work may be helpful to deepen the understanding of ferroptosis and provide a reference for clinical management and targeted treatment of GC.

## Discussion

As a recently discovered regulated cell death (RCD), ferroptosis has been proved to be closely related to tumor growth and progression, immune status, and anti-tumor effect. And its role in GC progression and treatment has gradually attracted people's attention (10, 38). In this study, we first recognized two clusters with prognostic differences by unsupervised clustering of the gene expression of FRGs. Afterward, DEGs with prognosis value among the two clusters were considered ferroptosis-specific genes and might be directly or indirectly regulated by ferroptosis events. Similar to the results of the first clustering, two ferroptosis subtypes were recognized based on the secondary clustering of these ferroptosis-specific genes.

Further studies have shown that the two subtypes also showed heterogeneity in sex and prognosis, mutation patterns, and immune cell infiltration. In addition, we established a scoring system set termed ferroptosis score to evaluate the ferroptosis levels of individual patients with GC. Further immunotherapy prediction also indicated that the ferroptosis score might be a promising immunotherapy marker. These results are helpful to deepen the understanding of ferroptosis and have a specific reference for the clinical management and outcome prediction of GC.

Sex is an essential factor in iron metabolism. The female' protective mechanisms against iron overload have been attributed to iron loss from female menstrual and sex hormones (39), whose roles in iron metabolism are not fully understood. The two subtypes show prominent clinical and gender characteristics. We observed that male GC patients with Subtype-B had the worst OS compared with Subtype-A. Previous studies have shown that AR is a potential therapeutic target for gastric cancer, predominantly female patients. The sex chromosome abnormalities and uncontrolled AR-related pathways may be significant causes of gender disparities (40). Thus further studies are warranted to pursue gender disparity in GC. The three sex hormone receptors (AR, ER, PR) were more expressed in Subtype-B than in Subtype-A, but the opposite was true in HER2. Auranofin (AUR) is a novel activator of hepcidin and ferroptosis via distinct mechanisms, and it has sex-specific effects concerning alleviating iron overload in a mouse model of hereditary hemochromatosis (41). Estrogen reduced the ability of AUR to induce IL-6/hepcidin signaling in Huh7 cells, providing a mechanistic explanation for the ineffectiveness of AUR in female Hfe–/– mice (a mouse model of hemochromatosis) (41). Thus, a different strategy for regulating the ferroptosis of tumor cells considers sex factors that may benefit patients and improve prognosis. Considering the heterogeneity of ferroptosis-related gene expression individually, there was a need to quantify the ferroptosis profiles of a single tumor. Thus, a ferroptosis-related scoring system called the ferroptosis score was further constructed. In addition, the ferroptosis score was positively correlated with three sex hormone receptors (AR, ER, PR) and negatively correlated with HER2. This also suggests that the ferroptosis score can also be regulated by gender difference-related factors such as sex hormone receptors.

Mutation analysis showed that the top three in the two subtypes were TTN, TP53, MUC16. Tumor suppressor gene TP53 is the most common mutant gene in cancer (42). TP53 mutation can modulate the ability of p53 to promote apoptosis and ferroptosis (8). Gastric cancer lacks therapy options and lacks predictive biomarkers. TP53 mutations at an early stage have been demonstrated to play a key role in gastric epithelial tumorigenesis and be considered “driver” mutations. The p53 family isoforms are at the center of multiple pathways in GC, including HIF1A, Hippo/YAP, TGF-β, TNF-αand NF-kB (43). Nevertheless, whether TP53 gene mutation may represent a biomarker of tumor invasiveness or therapeutic response is still worth exploring. As for TTN and MUC16, the study of gastric cancer or ferroptosis is still blank.

Researchers identified that ferroptosis was related to the immune response process (44). In addition to survival and sex, we analyzed the association between ferroptosis and immune cell infiltration in GC. The degree of 28 kinds of immune cell (26, 45, 46) infiltration in the two ferroptosis-related subtypes was significantly different. Moreover, the ferroptosis score had the strongest positive correlation with plasmacytoid dendritic cells and the strongest negative correlation with activated CD4 T cells. Cancer cells may die in response to anti-cancer therapies through regulated cell death programs, inhibiting or enhancing immunogenicity. In particular, the induction of immunogenic cancer cell death (ICD) is characterized by the emission of damage-associated molecular patterns (DAMPs) (47). Ferroptosis is a form of cell death, which is different from apoptosis and results from the accumulation of iron-dependent lipid peroxides (6, 12). It is unclear whether and how ferroptosis is involved in T cell immunity and cancer immunotherapy. Studies have shown that immunotherapy-activated CD8+ T cells enhance the specific lipid peroxidation of ferroptosis in tumor cells and that increased ferroptosis contributes to the anti-tumor efficacy of immunotherapy (48). In addition, T cells and interferon-gamma (IFN-γ) sensitize tumor cells to ferroptosis (48).

Moreover, the results of Tumor Immune Dysfunction and Exclusion (TIDE) showed that the low-ferroptosis score group had a higher response to immunotherapy in GC patients; this also validates the analysis results of TMB and MSI. Jiang et al. research had shown in melanoma patients that TIDE could more accurately predict the outcome of first-line anti-PD1 or anti-CTLA4 therapy than other biomarkers (such as PD-L1 level and mutation load) (35). All of these highlights the promising role of ferroptosis induction in GC immunotherapy.

In short, in clinical practice, the ferroptosis score could be used to comprehensively evaluate the ferroptosis-related patterns and their corresponding immune cell infiltration characterization within individual patients, further to guide the more effective clinical practice. We also demonstrated that the ferroptosis score could be utilized to assess patients' clinicopathological features, including MSI status, tumor mutation burden, immunotherapy response prediction, etc. We could also predict the drug sensitivity of adjuvant chemotherapy by ferroptosis score. More importantly, this study has yielded several novel insights for GC immunotherapy targeting ferroptosis-related specific genes to change the ferroptosis levels and reverse the negative immune cell infiltration characterization, providing novel ideas for promoting personalized GC immunotherapy in the future.

Nevertheless, the study also had several limitations. First, there is a lack of supervised clustering methods designed specifically for cancer genomics application, and unsupervised clustering is a choice for us at present. Second, the immunotherapy data of GC patients were not available now. The predictive performance of the ferroptosis score for GC needs to be further certified in the future. Third, we will continue to increase the sample size and the number of verification sets to improve the reliability and validity of the ferroptosis score in order to achieve more satisfactory results.

In conclusion, this study characterized two ferroptosis-related subtypes in gastric cancer, with different prognoses, gender characteristics, mutation characteristics, immune infiltration. Then we developed a risk model termed ferroptosis score to evaluate the ferroptosis level of individual tumors comprehensively; ferroptosis score demonstrated outstanding advantages in predicting prognosis and response to immunotherapy. Our study found gender differences in ferroptosis levels both in prognosis and immunotherapy. The ferroptosis score may be more suitable for males; of course, more verification research is necessary for the future.

## Data Availability Statement

Publicly available datasets were analyzed in this study. The data involved in this article could be downloaded directly in TCGA (https://portal.gdc.cancer.gov/) and GEO (https://www.ncbi.nlm.nih.gov/geo/) data sets (GSE62254(ACRG), GSE15459, GSE34942, GSE84437, GSE26899, GSE26901). Additional data for this study can be obtained from the corresponding author upon request.

## Author Contributions

JM and BL: conceptualization, formal analysis, and writing—original draft. XH, LW, and BL: data curation, methodology, and visualization. JM, YY, and LW: investigation. BL: project administration. XH, YY, and CS: software. BL and KC: supervision, validation, writing—review and editing, and funding acquisition. All authors have read and agreed to the published version of the manuscript.

## Funding

This work was supported by a grant from the National Natural Science Foundation of China (82073028, 81572445) to BL; National Natural Science Foundation of China (82172894) to KC; Tianjin Science and Technology Committee Foundation (Grant 20JCZXJC00090) to KC; National Key R&D Program of China (2017YFC0908300) to BL; Natural Science Foundation of Tianjin (16JCYBJC24700) to BL.

## Conflict of Interest

The authors declare that the research was conducted in the absence of any commercial or financial relationships that could be construed as a potential conflict of interest.

## Publisher's Note

All claims expressed in this article are solely those of the authors and do not necessarily represent those of their affiliated organizations, or those of the publisher, the editors and the reviewers. Any product that may be evaluated in this article, or claim that may be made by its manufacturer, is not guaranteed or endorsed by the publisher.

## Abbreviations

GC: gastric cancer
FRGs: Ferroptosis-related genes
GEO: Gene Expression Omnibus
TCGA: the Cancer Genome Atlas
OS: Overall survival
GSVA: Gene set variation analysis
ssGSEA: single-sample gene set enrichment analysis
PCA: Principal component analysis
TMB: Tumor mutational burden
MSI: Microsatellite instability
SMG: significantly mutated genes
TIDE: Tumor Immune Dysfunction and Exclusion
AR: androgen receptor
ER: the estrogen receptor
PR: progesterone receptor
HER2: human epidermal growth factor receptor 2.
