# Supplementary material for: Characterization of Two Ferroptosis Subtypes With Distinct Immune Infiltration and Gender Difference in Gastric Cancer
Source: Front Nutr. 2021 Dec 16;8:756193. doi: 10.3389/fnut.2021.756193 (PMC8716917; doi:10.3389/fnut.2021.756193)
Supplement: Supplementary file 1 [file Data_Sheet_1.PDF]

Figure S1. Unsupervised clustering for 60 ferroptosis-related genes.

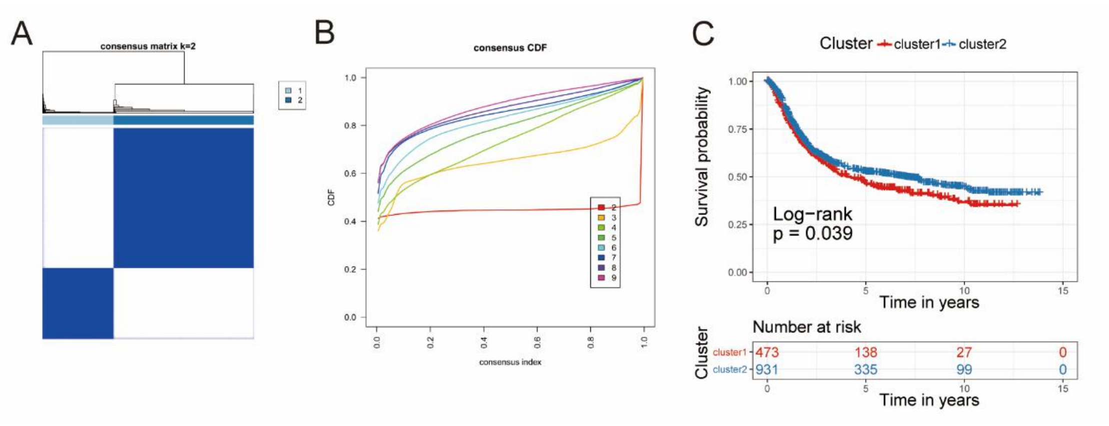

(A and **B)** The optimal number of clusters (K=2) was determined from cumulative distribution function (CDF) curves, and the classification effect is the best.

(C) Kaplan-Meier curves for survival prediction of patients in the two clusters.

Figure S2. Visualization of the contribution of two components of the PCA algorithm.

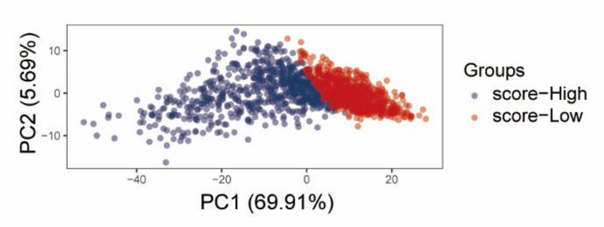

Figure S3. Validation of the predictive value of ferroptosis score in the aspect of 1 year AUC, 3 years AUC, and 5 years AUC via ROC curves analysis in the training set **(A)**, GSE26899 **(B)**, and GSE26901 **(C)**, respectively.

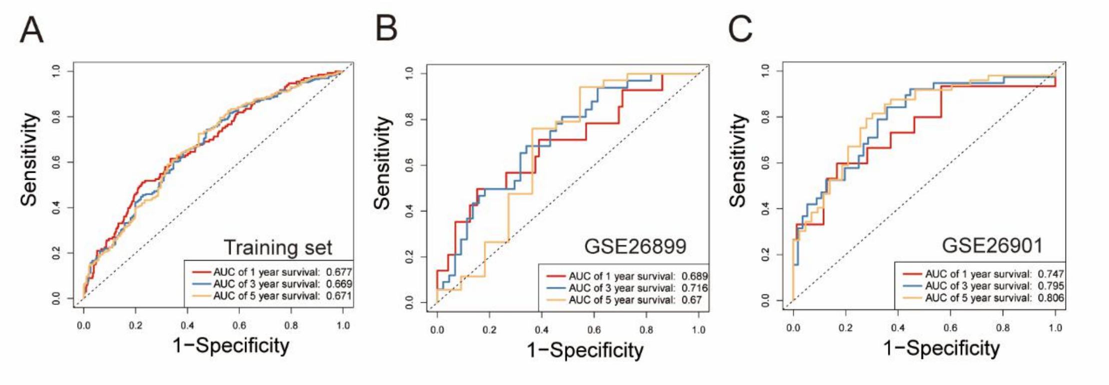

Figure S4. The Ferroptosis score among the molecular subtypes of gastric cancer in ACRG showed no significant difference (Kruskal-Wallis test, P=0.7).

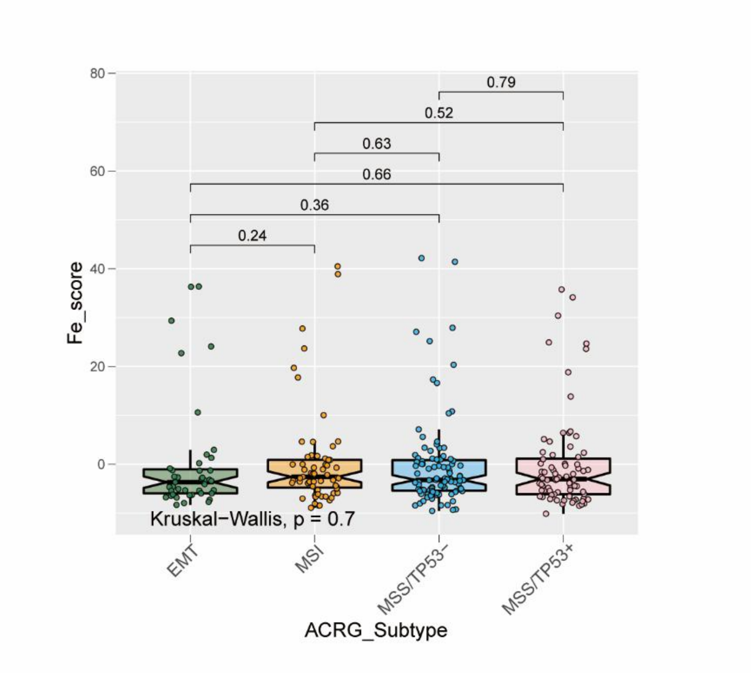

80
60
40
20
0
Fe_score
0.79
0.52
0.63
0.66
0.36
0.24
Kruskal−Wallis, p = 0.7
EMT
MSI
MSS/TP53−
MSS/TP53+
ACRG_Subtype
